# Crystal structure and Hirshfeld surface analysis of di­chlorido­(methanol-κ*O*)bis­(2-methyl­pyridine-κ*N*)copper(II)

**DOI:** 10.1107/S2056989020014036

**Published:** 2020-10-23

**Authors:** J. Prakasha Reddy

**Affiliations:** a Department of Chemistry, School of Sciences, Indrashil University, Rajpur, Gujarat, 382740, India

**Keywords:** crystal structure, hydrogen bonding, α-picoline, coordination chemistry

## Abstract

In the title complex, [CuCl_2_(C_6_H_7_N)_2_(CH_3_OH)], the copper atom is five-coordinated by two nitro­gen atoms of 2-methyl­pyridine ligands, two chloro ligands and an oxygen atom of the methanol mol­ecule, being in a tetra­gonal–pyramidal environment with N and Cl atoms forming the basal plane. In the crystal, complex mol­ecules related by the twofold rotation axis are joined into dimeric units by pairs of O—H⋯Cl hydrogen bonds. These dimeric units are assembled through C—H⋯Cl inter­actions into layers parallel to (001).

## Abstract 2

In the title complex, the Cu^II^ is in a tetra­gonal–pyramidal environment. The crystal structure features O—H⋯Cl and C—H⋯Cl inter­actions.

## Chemical context   

Both organic (from simple mol­ecules to peptides and proteins) and inorganic complexes have been known for more than a century and are central to modern chemistry because of their fascinating, aesthetic architectures and multiple applications (Gan *et al.*, 2011 ▸; Gellman, 1998 ▸; Thorat *et al.*, 2013 ▸; Vijayadas *et al.*, 2013 ▸; Ziach *et al.*, 2018 ▸). Recently, coordination compounds have been reported that find applications in fields such as catalysis, gas storage, separation technology and mol­ecular sensing (Mueller *et al.*, 2006 ▸; Wan *et al.*, 2006 ▸; Férey *et al.*, 2003 ▸; James, 2003 ▸; Eddaoudi *et al.*, 2002 ▸; Ruben *et al.*, 2005 ▸, Kitagawa *et al.*, 2004 ▸). There are many reports of coordination complexes where solvent mol­ecules are located in the voids of the crystal structure. However, reports describing the replacement of coordinated solvent mol­ecules with other mol­ecules are relatively scarce. As part of ongoing work in our laboratory, employing pyridine ligands in the preparation of various coordination networks (PrakashaReddy & Pedireddi, 2007 ▸), we have extended our work to the synthesis of other coordination networks. A literature survey revealed that coordination complex aqua­dichloro­bis­(2-methyl­pyridine)­copper(II) had been reported (Marsh *et al.*, 1982 ▸). Our inter­est was to see whether we could replace the coordinated water mol­ecule in the complex with other solvent mol­ecules such as methanol or ethanol *via* single-crystal-to-single-crystal transition (SCSCT) to investigate the structural changes. Although we could not succeed in SCSCT of the complex, we were successful in synthesizing the methanol-coordinated copper complex incorporating 2-methyl­pyridine as reported herein.
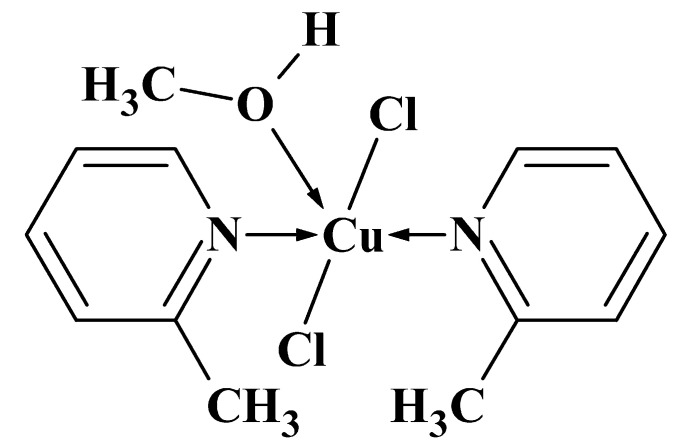


## Structural commentary   

**Figure 1:**
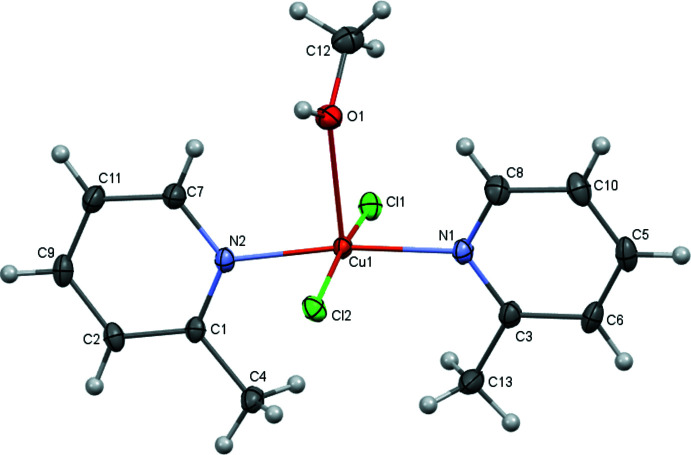
The mol­ecular structure of the title compound, showing the atom labelling and displacement ellipsoids drawn at the 50% probability level.

The title complex crystallizes in the monoclinic space group *C*2/*c* with one complex mol­ecule per asymmetric unit. Two nitro­gen atoms of 2-methyl­pyridine and two chloride ligands, which are *trans* to each other, form a rectangle around the copper atom, and its coordination is accomplished by the methanol oxygen atom, thus giving a tetra­gonal pyramid with the oxygen atom in the apical position (Fig. 1 ▸). The copper atom deviates by 0.161 (1) Å from the basal plane, and the angles around the copper atom are close to 90 and 180°. A plausible reason why the formation of a dimeric unit, as observed in [Cu(2-pic)_2_Cl_2_] (Marsh *et al.*, 1982 ▸), was precluded might be the presence of the coordinated methanol mol­ecule on one side of the coordination rectangle and the methyl groups on the other side. The methyl­pyridine rings form angles of 83.96 (8) and 85.70 (8)° with respect to the basal plane of the coordination polyhedron, thereby plausibly blocking the sixth coordination position at the copper atom. The Cu—O bond distance of 2.353 (2) Å is relatively short for an apical atom in typical copper(II) tetra­gonal–pyramidal structure, whereas the Cu—N bond lengths [Cu1—N1= 2.031 (2) Å, Cu1—N2 = 2.017 (2) Å] agree well with those reported for related structures (Wang *et al.*, 2006 ▸; Gong *et al.*, 2009 ▸; Hu & Zhang, 2010 ▸; Li, 2011 ▸; Sun *et al.*, 2013 ▸; Sanram *et al.*, 2016 ▸).

## Supra­molecular features and Hirshfeld surface analysis   

**Figure 2:**
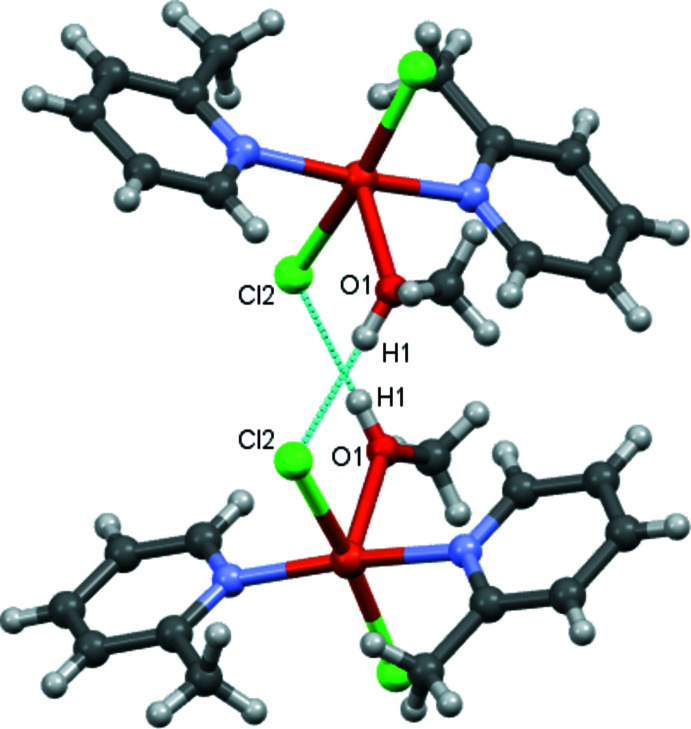
The O—H⋯Cl inter­actions between two mol­ecules in the crystal of the title compound. The mol­ecules are related by the symmetry operation −*x* + 1, *y*, −*z* + 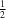.

**Figure 3:**
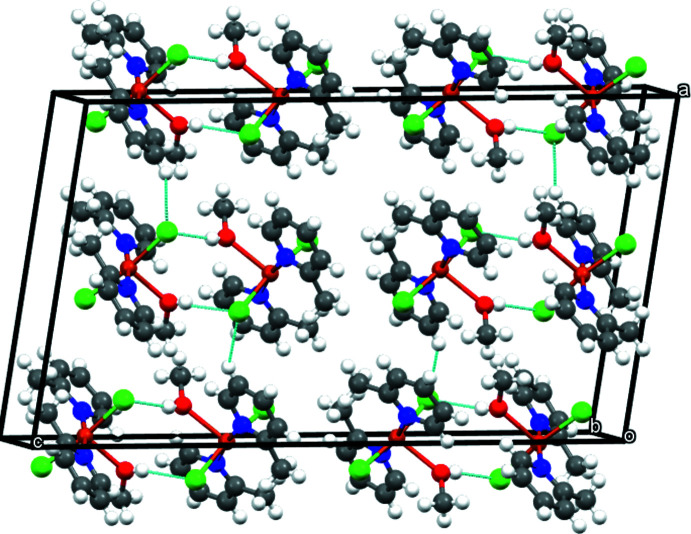
A general view of the crystal packing of the title compound along the *b-*axis direction with inter­molecular contacts shown as dashed lines.

Complex mol­ecules related by the twofold rotation axis are connected by pairs of O—H⋯Cl inter­actions (Table 1 ▸) involving the apical methanol ligand of one complex and a chloride ligand of the other, thus forming dimers (Fig. 2 ▸). The O⋯Cl and H⋯Cl distances and associated O—H⋯Cl angle lie within the ranges observed for other O—H⋯Cl inter­actions reported in the literature (Veal *et al.*, 1972 ▸; Taylor, 2016 ▸; Ristić *et al.*, 2020 ▸; Estes *et al.*, 1976 ▸). These dimers are further connected through C—H⋯Cl inter­actions, generating layers parallel to (001) (Fig. 3 ▸, Table 1 ▸).

**Figure 4:**
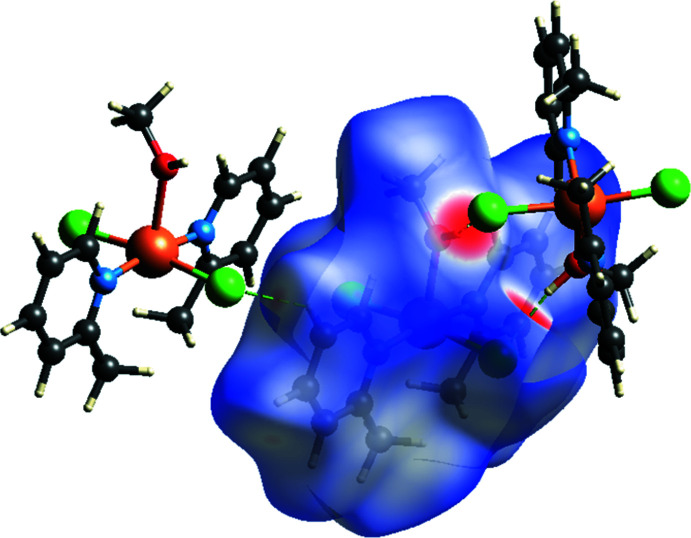
Hirshfeld surface mapped over *d*_norm_ highlighting the regions of O—H⋯Cl and C—H⋯Cl inter­molecular contacts.

**Figure 5:**
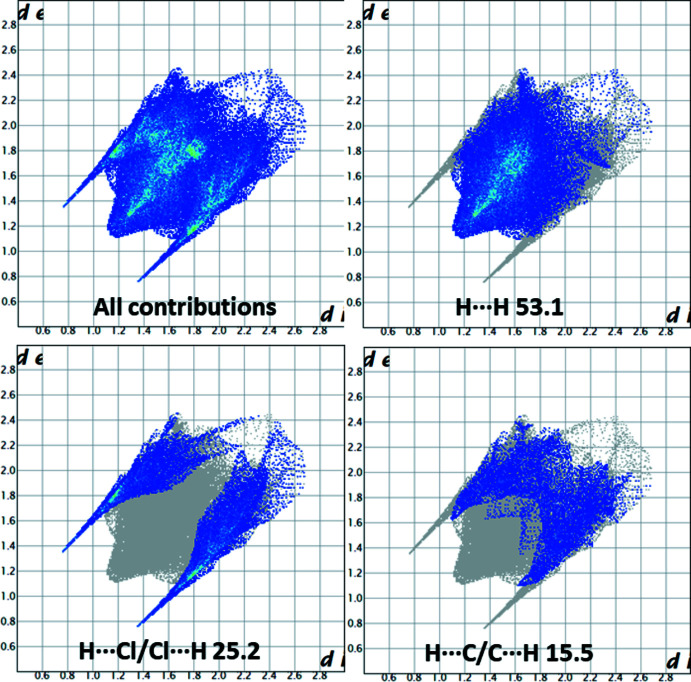
The full two-dimensional fingerprint plot for the title compound and those delineated into H⋯H (53.1%), Cl⋯H/H⋯Cl (25.2%) and C⋯H/H⋯C (15.5%) contacts.

A Hirshfeld surface analysis was performed and two-dimensional fingerprint plots were prepared using *Crystal Explorer17* (Turner *et al.*, 2017 ▸) to further investigate the inter­molecular inter­actions in the title structure. The Hirshfeld surface mapped over *d*_norm_ with corresponding colours representing inter­molecular inter­actions is shown in Fig. 4 ▸. The red spots on the surface correspond to the O—H⋯Cl, C—H⋯Cl and C—H⋯O inter­actions (Table 1 ▸). The two-dimensional fingerprint plots (McKinnon *et al.*, 2007 ▸) are shown in Fig. 5 ▸. Weak van der Waals H⋯H contacts make the largest contribution (53.1%) to the Hirshfeld surface. The two-dimensional fingerprint plot shows two spikes that correspond to H⋯Cl/Cl⋯H (25.2%) inter­actions, which highlight the hydrogen bonds between adjacent mol­ecules. The C⋯H/H⋯C (15.5%) inter­actions also appear as two spikes. These inter­actions play a crucial role in the overall cohesion of the crystal packing.

## Database survey   

A search of the Cambridge Structural Database (CSD, Version 5.40, update of August 2019; Groom *et al.*, 2016 ▸) revealed three closely related complexes: di­chloro­bis­(2-methyl­pyridine)­copper(II) (refcode CMPYCU01; Marsh *et al.*, 1982 ▸), aqua­dichloro­bis­(2-methyl­pyridine)­copper(II) (BIJWUM; Marsh *et al.*, 1982 ▸) and bis­(iso­thio­cyanato)­methanolbis­(2-methyl­pyridine)­copper(II) (ABOSIW; Handy *et al.*, 2017 ▸). Structures CMPYCU01 and BIJWUM display dimeric arrangements of the complex mol­ecules arising from C—H⋯Cl and O—H⋯Cl inter­actions, respectively, while in the copper(II) thio­cyanate complex ABOSIW, the three-dimensional network is formed as a result of O—H⋯S, C—H⋯S and C—H⋯C inter­actions.

## Synthesis and crystallization   

2-Methyl­pyridine and anhydrous copper(II) chloride were obtained from Aldrich, and HPLC grade methanol was used for reaction. Anhydrous copper(II) chloride (0.675 g, 0.005 mol) was dissolved in 15 ml of methanol. To this solution, 2-methyl­pyridine (0.93 g, 0.01 mol) dissolved in 15 mL of methanol was added. The resulting mixture was stirred for *ca* 40 min. at room temperature and filtered to remove the greenish precipitate. The blue filtrate was then allowed to stand at room temperature for a few hours, before being filtered and left at room temperature for crystallization. A mixture of dark-blue crystals of different sizes was obtained after 24 h.

## Refinement   

Crystal data, data collection and structure refinement details are summarized in Table 2 ▸. All H atoms were located in a difference map. The C-bound H atoms were placed in calculated positions with C—H = 0.93-0.96 Å and refined as riding, whereas the coordinates of O-bound H atom were freely refined. All hydrogen atoms were refined with fixed isotropic displacement parameters [*U*_iso_(H) = 1.2–1.5*U*_eq_(C,O)]

## Supplementary Material

Crystal structure: contains datablock(s) I. DOI: 10.1107/S2056989020014036/yk2140sup1.cif

Structure factors: contains datablock(s) I. DOI: 10.1107/S2056989020014036/yk2140Isup2.hkl

CCDC reference: 1997065

Additional supporting information:  crystallographic information; 3D view; checkCIF report

## Figures and Tables

**Table 1:** Hydrogen-bond geometry (Å, °)

*D*—H⋯*A*	*D*—H	H⋯*A*	*D*⋯*A*	*D*—H⋯*A*
O1—H1⋯Cl2^i^	0.75 (3)	2.37 (3)	3.1033 (16)	169 (3)
C7—H7⋯O1	0.93	2.46	3.148 (3)	130
C8—H8⋯O1	0.93	2.34	3.036 (3)	131
C11—H11⋯Cl2^ii^	0.93	2.83	3.624 (2)	143

Symmetry codes: (i) 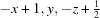; (ii) 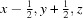.

**Table 2:** Experimental details

Crystal data	
Chemical formula	[CuCl_2_(C_6_H_7_N)_2_(CH_4_O)]
*M*_r_	352.73
Crystal system, space group	Monoclinic, *C*2/*c*
Temperature (K)	120
*a*, *b*, *c* (Å)	14.4554 (4), 8.5865 (2), 24.8055 (8)
β (°)	99.209 (3)
*V* (Å^3^)	3039.22 (16)
*Z*	8
Radiation type	Mo *K*α
μ (mm^−1^)	1.78
Crystal size (mm)	0.21 × 0.16 × 0.11

Data collection	
Diffractometer	Agilent XCalibur diffractometer
Absorption correction	Multi-scan (*CrysAlis PRO*; Rigaku OD, 2018 ▸)
*T*_min_, *T*_max_	0.549, 1.000
No. of measured, independent and observed [*I* > 2σ(*I*)] reflections	15974, 5137, 4251
*R*_int_	0.040
(sin θ/λ)_max_ (Å^−1^)	0.758

Refinement	
*R*[*F* ^2^ > 2σ(*F* ^2^)], *wR*(*F* ^2^), *S*	0.038, 0.081, 1.13
No. of reflections	5137
No. of parameters	178
H-atom treatment	H atoms treated by a mixture of independent and constrained refinement
Δρ_max_, Δρ_min_ (e Å^−3^)	0.54, −0.51

Computer programs: *CrysAlis CCD* and *CrysAlis RED* (Oxford Diffraction, 2009 ▸), *SHELXT* (Sheldrick, 2015*a* ▸), *SHELXL* (Sheldrick, 2015*b* ▸) and *OLEX2* (Dolomanov *et al.*, 2009 ▸).

## supplementary crystallographic information

### Crystal data

**Table:**

[CuCl_2_(C_6_H_7_N)_2_(CH_4_O)]	*F*(000) = 1448
*M**_r_* = 352.73	*D*_x_ = 1.542 Mg m^−^^3^
Monoclinic, *C*2/*c*	Mo *K*α radiation, λ = 0.71073 Å
*a* = 14.4554 (4) Å	Cell parameters from 4621 reflections
*b* = 8.5865 (2) Å	θ = 3.1–32.0°
*c* = 24.8055 (8) Å	µ = 1.78 mm^−^^1^
β = 99.209 (3)°	*T* = 120 K
*V* = 3039.22 (16) Å^3^	Block, blue
*Z* = 8	0.21 × 0.16 × 0.11 mm

### Data collection

**Table:**

Agilent XCalibur diffractometer	4251 reflections with *I* > 2σ(*I*)
Detector resolution: 16.1511 pixels mm^-1^	*R*_int_ = 0.040
ω scans	θ_max_ = 32.6°, θ_min_ = 2.8°
Absorption correction: multi-scan (CrysAlisPro; Rigaku OD, 2018)	*h* = −20→21
*T*_min_ = 0.549, *T*_max_ = 1.000	*k* = −12→11
15974 measured reflections	*l* = −37→37
5137 independent reflections	

### Refinement

**Table:**

Refinement on *F*^2^	Primary atom site location: dual
Least-squares matrix: full	Hydrogen site location: mixed
*R*[*F*^2^ > 2σ(*F*^2^)] = 0.038	H atoms treated by a mixture of independent and constrained refinement
*wR*(*F*^2^) = 0.081	*w* = 1/[σ^2^(*F*_o_^2^) + (0.0199*P*)^2^ + 4.4488*P*] where *P* = (*F*_o_^2^ + 2*F*_c_^2^)/3
*S* = 1.13	(Δ/σ)_max_ = 0.001
5137 reflections	Δρ_max_ = 0.54 e Å^−^^3^
178 parameters	Δρ_min_ = −0.51 e Å^−^^3^
0 restraints	

### Special details

**Table:**

Geometry. All esds (except the esd in the dihedral angle between two l.s. planes) are estimated using the full covariance matrix. The cell esds are taken into account individually in the estimation of esds in distances, angles and torsion angles; correlations between esds in cell parameters are only used when they are defined by crystal symmetry. An approximate (isotropic) treatment of cell esds is used for estimating esds involving l.s. planes.

### Fractional atomic coordinates and isotropic or equivalent isotropic displacement parameters (Å^2^)

**Table:**

	*x*	*y*	*z*	*U*_iso_*/*U*_eq_	
Cu1	0.50986 (2)	0.75598 (3)	0.37062 (2)	0.01272 (6)	
Cl1	0.41953 (3)	0.66088 (6)	0.43059 (2)	0.01703 (10)	
Cl2	0.61677 (3)	0.86629 (5)	0.32152 (2)	0.01706 (10)	
O1	0.40656 (10)	0.69534 (18)	0.29006 (6)	0.0192 (3)	
H1	0.408 (2)	0.738 (3)	0.2640 (12)	0.029*	
N2	0.45788 (11)	0.97058 (19)	0.38001 (7)	0.0145 (3)	
N1	0.57721 (11)	0.54847 (19)	0.37032 (7)	0.0149 (3)	
C1	0.49779 (13)	1.0690 (2)	0.41912 (8)	0.0141 (3)	
C2	0.45939 (14)	1.2154 (2)	0.42581 (8)	0.0168 (4)	
H2	0.488544	1.282802	0.452631	0.020*	
C3	0.64638 (14)	0.5067 (2)	0.41102 (9)	0.0175 (4)	
C4	0.58446 (14)	1.0157 (2)	0.45567 (8)	0.0188 (4)	
H4A	0.571444	0.921107	0.473669	0.028*	
H4B	0.604355	1.094501	0.482496	0.028*	
H4C	0.633209	0.997353	0.434317	0.028*	
C5	0.66783 (15)	0.2646 (2)	0.36638 (9)	0.0220 (4)	
H5	0.698699	0.170012	0.364995	0.026*	
C6	0.69292 (14)	0.3650 (2)	0.40943 (9)	0.0216 (4)	
H6	0.740983	0.338329	0.437494	0.026*	
C7	0.37914 (14)	1.0160 (2)	0.34693 (9)	0.0201 (4)	
H7	0.351990	0.948582	0.319564	0.024*	
C8	0.55316 (15)	0.4492 (2)	0.32849 (8)	0.0190 (4)	
H8	0.505512	0.477838	0.300461	0.023*	
C9	0.37781 (15)	1.2600 (2)	0.39241 (9)	0.0192 (4)	
H9	0.350701	1.356328	0.396982	0.023*	
C10	0.59622 (16)	0.3063 (2)	0.32532 (9)	0.0220 (4)	
H10	0.577215	0.239722	0.296070	0.026*	
C11	0.33724 (15)	1.1589 (3)	0.35209 (9)	0.0223 (4)	
H11	0.282686	1.186387	0.328834	0.027*	
C12	0.32370 (15)	0.6023 (3)	0.28385 (9)	0.0231 (4)	
H12A	0.326320	0.525448	0.256034	0.035*	
H12B	0.269827	0.667541	0.273541	0.035*	
H12C	0.319211	0.551434	0.317792	0.035*	
C13	0.67215 (16)	0.6175 (3)	0.45740 (10)	0.0271 (5)	
H13A	0.691508	0.714806	0.443729	0.041*	
H13B	0.722622	0.574607	0.482943	0.041*	
H13C	0.618872	0.634370	0.475334	0.041*	

### Atomic displacement parameters (Å^2^)

**Table:**

	*U*^11^	*U*^22^	*U*^33^	*U*^12^	*U*^13^	*U*^23^
Cu1	0.01378 (11)	0.01059 (11)	0.01375 (11)	0.00289 (8)	0.00206 (8)	−0.00104 (8)
Cl1	0.0200 (2)	0.0157 (2)	0.0161 (2)	0.00216 (17)	0.00497 (17)	0.00003 (17)
Cl2	0.0182 (2)	0.0149 (2)	0.0192 (2)	−0.00028 (16)	0.00627 (17)	−0.00322 (17)
O1	0.0226 (7)	0.0190 (7)	0.0152 (7)	−0.0020 (6)	0.0008 (6)	0.0024 (6)
N2	0.0145 (7)	0.0122 (7)	0.0175 (8)	0.0024 (6)	0.0047 (6)	0.0004 (6)
N1	0.0152 (7)	0.0133 (7)	0.0168 (8)	0.0020 (6)	0.0045 (6)	−0.0011 (6)
C1	0.0168 (8)	0.0135 (8)	0.0128 (8)	0.0014 (7)	0.0049 (7)	0.0018 (7)
C2	0.0226 (9)	0.0128 (9)	0.0165 (9)	0.0015 (7)	0.0075 (8)	−0.0011 (7)
C3	0.0157 (9)	0.0155 (9)	0.0217 (10)	0.0024 (7)	0.0041 (7)	0.0004 (8)
C4	0.0216 (9)	0.0175 (9)	0.0164 (9)	0.0042 (8)	0.0005 (8)	−0.0029 (8)
C5	0.0252 (10)	0.0135 (9)	0.0304 (11)	0.0057 (8)	0.0138 (9)	0.0027 (8)
C6	0.0186 (9)	0.0196 (10)	0.0264 (11)	0.0077 (8)	0.0030 (8)	0.0034 (8)
C7	0.0178 (9)	0.0175 (9)	0.0237 (10)	0.0031 (7)	−0.0009 (8)	−0.0033 (8)
C8	0.0235 (10)	0.0190 (10)	0.0154 (9)	0.0031 (8)	0.0055 (8)	−0.0020 (8)
C9	0.0233 (10)	0.0138 (9)	0.0225 (10)	0.0058 (7)	0.0094 (8)	0.0022 (8)
C10	0.0293 (11)	0.0163 (9)	0.0222 (10)	0.0021 (8)	0.0101 (9)	−0.0044 (8)
C11	0.0198 (9)	0.0205 (10)	0.0252 (11)	0.0076 (8)	−0.0007 (8)	−0.0007 (8)
C12	0.0202 (10)	0.0289 (11)	0.0202 (10)	0.0001 (8)	0.0033 (8)	−0.0023 (9)
C13	0.0236 (10)	0.0235 (11)	0.0303 (12)	0.0082 (9)	−0.0081 (9)	−0.0069 (9)

### Geometric parameters (Å, º)

**Table:**

Cu1—Cl1	2.2818 (5)	C4—H4C	0.9600
Cu1—Cl2	2.3175 (5)	C5—H5	0.9300
Cu1—O1	2.3534 (15)	C5—C6	1.375 (3)
Cu1—N2	2.0174 (16)	C5—C10	1.378 (3)
Cu1—N1	2.0310 (16)	C6—H6	0.9300
O1—H1	0.75 (3)	C7—H7	0.9300
O1—C12	1.427 (3)	C7—C11	1.383 (3)
N2—C1	1.345 (2)	C8—H8	0.9300
N2—C7	1.350 (3)	C8—C10	1.384 (3)
N1—C3	1.351 (3)	C9—H9	0.9300
N1—C8	1.345 (3)	C9—C11	1.382 (3)
C1—C2	1.395 (3)	C10—H10	0.9300
C1—C4	1.496 (3)	C11—H11	0.9300
C2—H2	0.9300	C12—H12A	0.9600
C2—C9	1.382 (3)	C12—H12B	0.9600
C3—C6	1.394 (3)	C12—H12C	0.9600
C3—C13	1.494 (3)	C13—H13A	0.9600
C4—H4A	0.9600	C13—H13B	0.9600
C4—H4B	0.9600	C13—H13C	0.9600
			
Cl1—Cu1—Cl2	171.17 (2)	C6—C5—H5	120.5
Cl1—Cu1—O1	97.06 (4)	C6—C5—C10	118.99 (19)
Cl2—Cu1—O1	91.73 (4)	C10—C5—H5	120.5
N2—Cu1—Cl1	89.37 (5)	C3—C6—H6	120.0
N2—Cu1—Cl2	88.82 (5)	C5—C6—C3	120.0 (2)
N2—Cu1—O1	95.90 (6)	C5—C6—H6	120.0
N2—Cu1—N1	171.61 (7)	N2—C7—H7	118.7
N1—Cu1—Cl1	90.74 (5)	N2—C7—C11	122.6 (2)
N1—Cu1—Cl2	89.79 (5)	C11—C7—H7	118.7
N1—Cu1—O1	92.42 (6)	N1—C8—H8	118.6
Cu1—O1—H1	121 (2)	N1—C8—C10	122.9 (2)
C12—O1—Cu1	128.53 (13)	C10—C8—H8	118.6
C12—O1—H1	109 (2)	C2—C9—H9	120.6
C1—N2—Cu1	122.19 (13)	C2—C9—C11	118.84 (19)
C1—N2—C7	118.63 (17)	C11—C9—H9	120.6
C7—N2—Cu1	119.16 (14)	C5—C10—C8	118.7 (2)
C3—N1—Cu1	121.89 (13)	C5—C10—H10	120.7
C8—N1—Cu1	119.60 (13)	C8—C10—H10	120.7
C8—N1—C3	118.50 (17)	C7—C11—H11	120.6
N2—C1—C2	121.29 (18)	C9—C11—C7	118.87 (19)
N2—C1—C4	117.75 (17)	C9—C11—H11	120.6
C2—C1—C4	120.96 (18)	O1—C12—H12A	109.5
C1—C2—H2	120.1	O1—C12—H12B	109.5
C9—C2—C1	119.71 (19)	O1—C12—H12C	109.5
C9—C2—H2	120.1	H12A—C12—H12B	109.5
N1—C3—C6	120.91 (19)	H12A—C12—H12C	109.5
N1—C3—C13	118.02 (17)	H12B—C12—H12C	109.5
C6—C3—C13	121.06 (19)	C3—C13—H13A	109.5
C1—C4—H4A	109.5	C3—C13—H13B	109.5
C1—C4—H4B	109.5	C3—C13—H13C	109.5
C1—C4—H4C	109.5	H13A—C13—H13B	109.5
H4A—C4—H4B	109.5	H13A—C13—H13C	109.5
H4A—C4—H4C	109.5	H13B—C13—H13C	109.5
H4B—C4—H4C	109.5		
			
Cu1—N2—C1—C2	−178.36 (14)	C1—C2—C9—C11	−1.5 (3)
Cu1—N2—C1—C4	1.1 (2)	C2—C9—C11—C7	0.6 (3)
Cu1—N2—C7—C11	177.53 (17)	C3—N1—C8—C10	−0.1 (3)
Cu1—N1—C3—C6	−178.70 (15)	C4—C1—C2—C9	−178.23 (18)
Cu1—N1—C3—C13	0.6 (3)	C6—C5—C10—C8	1.1 (3)
Cu1—N1—C8—C10	179.63 (16)	C7—N2—C1—C2	0.0 (3)
N2—C1—C2—C9	1.2 (3)	C7—N2—C1—C4	179.42 (18)
N2—C7—C11—C9	0.5 (3)	C8—N1—C3—C6	1.0 (3)
N1—C3—C6—C5	−0.9 (3)	C8—N1—C3—C13	−179.70 (19)
N1—C8—C10—C5	−1.0 (3)	C10—C5—C6—C3	−0.2 (3)
C1—N2—C7—C11	−0.9 (3)	C13—C3—C6—C5	179.8 (2)

### Hydrogen-bond geometry (Å, º)

**Table:**

*D*—H···*A*	*D*—H	H···*A*	*D*···*A*	*D*—H···*A*
O1—H1···Cl2^i^	0.75 (3)	2.37 (3)	3.1033 (16)	169 (3)
C7—H7···O1	0.93	2.46	3.148 (3)	130
C8—H8···O1	0.93	2.34	3.036 (3)	131
C11—H11···Cl2^ii^	0.93	2.83	3.624 (2)	143

Symmetry codes: (i) −*x*+1, *y*, −*z*+1/2; (ii) *x*−1/2, *y*+1/2, *z*.
